# Opening Access to Cell Biology

**DOI:** 10.1371/journal.pbio.0030426

**Published:** 2005-12-01

**Authors:** Caitlin Sedwick

## Abstract

*PLoS Biology* is pleased to present a collection of articles focused on cell biology.

In October 2003, the inaugural issue of *PLoS Biology* hit desktops everywhere—demonstrating the power of open-access scientific publishing. Our hopes for our flagship journal were borne out with the publication of several articles (including [ 1, 2]) that captured the public and scientific imagination, triggering both widespread press coverage and impressive numbers of downloads and citations since their publication.

We were obviously pleased by this response, but we were not surprised by it. Papers published by *PLoS Biology* are distributed under the Creative Commons Open Access license, which means they are immediately available online, free of charge, with no restriction on their use (as long as the original authors receive appropriate attribution). As a result, our papers are more accessible [ 3], more usable [ 4, 5], and, given initial evidence, more likely to be *used* than papers published under the conventional licensing schemes employed by subscription-only, pay-for-access journals.

This initial success of *PLoS Biology* is a practical validation of our raison d'être: to make primary research materials a public resource. The public (and public interests) pays for most of it, you do the work and write the papers, and everyone—you, your colleagues, your students, patients, and neighbors—should be able to use and benefit from the efforts they helped underwrite. The U.S. government and major funding agencies worldwide agree; since the launch of *PLoS Biology*, the NIH has started asking its funded researchers to deposit their work in public databases [ 6], while institutions such as the Wellcome Trust [ 7] and Howard Hughes [ 8] have enacted policies supporting open access to the scientific literature. In response, the scientific publishing industry is beginning to accommodate demands for open access. For example, *The Journal of Cell Biology* is complying with NIH requirements by making its articles freely available six months after publication [ 9]. Still others, such as *Proceedings of the National Academy of Sciences*, now offer a hybrid publication model, which allows authors to choose to have their work made available immediately upon publication [ 10].

We made a strategic decision that positioning *PLoS Biology* in direct competition with the most prestigious journals out there would improve public access to scientific material. This strategy is paying off. Although *PLoS Biology* is still in its infancy, we are now ranked as the leading general biology journal (with a preliminary ISI impact factor of 13.9), and we remain committed to publishing the best research in the biological sciences. Over the two years that we've been in business, we've accumulated a catalog of papers from across the spectrum of biological research: from molecular to whole organism, from empirical to theoretical, and from established fields such as cell biology and ecology to recently emerging disciplines such as systems biology, bioinformatics, and proteomics.

In the *PLoS Biology* Cell Biology collection, available at http://www.plosbiology.org/cellbiology/, we highlight some of the seminal work we've published in cell biology and related fields, including primary research papers on stem cells, hair development, endocytosis and exocytosis, cohesins, and the cytoskeleton. Some of the authors of these papers chose to publish in *PLoS Biology* because they believe in open access; others decided to do it because they just wanted to publish in a great journal.

You'll also find other types of articles in this supplement. Each research paper we publish is accompanied by a lay synopsis written by a science writer and approved by the paper's authors. Synopses add value to work published in *PLoS Biology* by making the research more accessible to students, scientists from other fields, and the public at large. Primers serve as an introduction to a complicated subject: the three primers in this supplement explore stem cell biology; for example, one accompanied the recent publication of Rendl et al.'s manuscript on hair follicle development. These primers are already being used in classrooms and lectures around the world. Finally, you'll find an essay written by one of our editorial board members—Dr. Tom Misteli—who describes his viewpoint on an important direction in aging research.

We hope you find the research papers and other journal offerings in this collection interesting and useful. This collection is by no means the complete cell biology content of our journal, but we think these materials do a great job of demonstrating the value that *PLoS Biology* provides, which is a direct result of the efforts and commitment of our academic advisory board members and our authors. We hope that you will be inspired to read *PLoS Biology* regularly (you can sign up for regular eTOC alerts at http://admin.co.allenpress.com/admin/plosonline/webusers/public/form). We also hope that when it comes time to publish your best work, you'll consider PLoS. You'll be in good company!
